# Acceptability of single-dose HPV vaccination schedule among health-care professionals in Kenya: a mixed-methods study

**DOI:** 10.1093/jncimonographs/lgae031

**Published:** 2024-11-12

**Authors:** Grace Umutesi, Bryan J Weiner, Lynda Oluoch, Elizabeth Bukusi, Maricianah Onono, Betty Njoroge, Lucy Mecca, Kenneth Ngure, Nelly R Mugo, Ruanne V Barnabas

**Affiliations:** Department of Global Health, University of Washington, Seattle, WA, USA; Department of Global Health, University of Washington, Seattle, WA, USA; Center for Clinical Research, Kenya Medical Research Institute, Nairobi, Kenya; Department of Global Health, University of Washington, Seattle, WA, USA; Center for Clinical Research, Kenya Medical Research Institute, Nairobi, Kenya; Center for Clinical Research, Kenya Medical Research Institute, Nairobi, Kenya; Center for Clinical Research, Kenya Medical Research Institute, Nairobi, Kenya; School of Public Health, Jomo Kenyatta University of Agriculture and Technology, Nairobi, Kenya; Department of Global Health, University of Washington, Seattle, WA, USA; Ministry of Health, Kenya; Department of Global Health, University of Washington, Seattle, WA, USA; Center for Clinical Research, Kenya Medical Research Institute, Nairobi, Kenya; Division of Infectious Diseases, Massachusetts General Hospital, Boston, MA, USA; Department of Medicine, Harvard Medical School, Boston, MA, USA

## Abstract

**Background:**

The World Health Organization recommends a single-dose human papillomavirus (HPV) vaccination schedule for girls and boys to accelerate progress toward cervical cancer elimination. We applied the Theoretical Framework of Acceptability (TFA) within the context of HPV vaccination to assess the acceptability of a single-dose schedule among health-care professionals in Kenya.

**Methods:**

A REDCap survey was developed using relevant Theoretical Framework of Acceptability domains and validated with health-care professionals. Descriptive analyses and multivariate Poisson regression were conducted to assess factors associated with increased acceptability. Free-text responses were analyzed using a rapid qualitative approach, and findings were presented using a joint display.

**Results:**

Among 385 responses, 74.2% of health-care professionals were female and 48.6% were nurses. On average, respondents had been in their position for 60 months, and one-third (33.2%) were based at level-4 facilities. The majority (75.84%) thought that giving a single-dose of the HPV vaccine to adolescent girls and young women was either acceptable or very acceptable. Qualitative findings highlighted that lack of information was the underlying reason for health-care professionals who were resistant, and most clinicians thought that a singled-dose schedule was less burdensome to clinicians and patients. Hospital directors had a non–statistically significantly lower acceptability likelihood than nurses (incident rate ratio = 0.93, 95% confidence interval = 0.45 to 1.71) and health-care professionals at urban facilities had a non–statistically significantly lower acceptability likelihood than clinicians in rural facilities (incident rate ratio = 0.97, 95% confidence interval = 0.83 to 1.13).

**Conclusion:**

Although not statistically significant, predictors of increased acceptability provide information to tailor strategies to increase HPV vaccination coverage and accelerate progress toward cervical cancer elimination.

Cervical cancer is the fourth-leading cause of death among women. Low- and middle-income countries (LMICs), where more than 85% of cervical cancer cases occur, are disproportionately impacted (1-3). Human papillomavirus (HPV) infections are the primary cause of cervical cancer. HPV vaccines have high efficacy, preventing persistent HPV infections, a precursor for dysplastic lesions and cervical cancer (4). Despite this, HPV vaccination coverage remains low in many LMICs, including in sub-Saharan Africa (5,6). Health system challenges, stigma, fear of side effects, misinformation, and inadequate health education have been identified as barriers to HPV vaccination uptake in sub-Saharan Africa (7). In Kenya specifically, less than one-third of girls aged 9 to 14 years had received 1 dose, and only 16% had received 2 doses in 2020 (8). The strength of evidence for single-dose HPV vaccine effectiveness is substantial and irrefutable (9). In a randomized trial in Kenya, a single-dose of HPV vaccine was highly efficacious, with a vaccine efficacy of 97.5% for the bivalent vaccine and 98.8% for the nonavalent vaccine to prevent incident persistent HPV-16/18 infection (10-12). Similar results highlighting the efficacy of a single-dose of HPV vaccine in providing protection against persistent HPV-16/18 infections for more than a decade after vaccination have been reported in other settings (13-16). At the population level, HPV vaccination of adolescent girls and young women conferred high protection against cervical cancer, irrespective of the number of doses (17). These findings support the single-dose HPV vaccination schedule recommendations from the World Health Organization to reach adolescent girls and young women and accelerate progress toward cervical cancer elimination (18). Evidence is needed to optimize HPV vaccination delivery approaches, especially in settings with low HPV vaccine coverage.

Health-care professionals play a vital role as communicators and enablers of immunization. A systematic review of studies on the knowledge, awareness, and acceptability of the HPV vaccine reported that in South Africa, 46% of women aged 18-44 years were more likely to be vaccinated based on their clinician’s recommendation. This recommendation was rated higher (ie, more important) than effectiveness or cost (19). Similar findings were highlighted in a recent study conducted among adults who had children aged 12 to 14 years who were seeking care at the Kenyatta National Hospital (20). In this study, 86% of participants reported that a doctor’s recommendation was the main reason for their willingness to have their daughter vaccinated. In addition, health-care professionals’ perception and acceptability of HPV vaccination affected their recommendation regarding the vaccine, which in turn affected vaccine uptake. Health-care professionals who have vaccine confidence, driven by correct knowledge of the vaccine and the disease it prevents, are more likely to recommend the vaccine to patients. Their recommendations are known to have a considerable impact on patient uptake across vaccines and populations (21). Although the concept of acceptability has been defined differently in implementation research, these definitions are closely related to the definition of acceptability in the Theorical Framework of Acceptability (TFA) (22-26). The TFA has the advantage of allowing assessment of the acceptability of an evidence-based intervention before, during, and after the delivery of that intervention (22,25). In addition, the TFA was developed using both inductive (empirical) and deductive (theorical) approaches. Nevertheless, the TFA, as several other implementation research frameworks, was informed by insights from high-income countries. Most implementation science theories and frameworks remain untested in LMICs (27). It is crucial to assess the relevance of implementation science frameworks in guiding implementation theories and frameworks for LMICs.

To address these gaps and generate evidence to inform HPV vaccine delivery, we assessed the application of the TFA in the context of HPV vaccination in Kenya and evaluated the acceptability and predictors of acceptability of the single-dose HPV vaccination schedule among health-care professionals in Kenya.

## Methods

This study was nested within the KEN SHE Study on HPV-vaccine Efficacy (ClinicalTrials.gov indentifier NCT03675256). KEN SHE is a randomized, multicenter, double-blind, 3-arm, controlled trial that tested the efficacy of single-dose bivalent (HPV-16/18) and single-dose nonavalent (HPV-16, 18, 31, 33, 45, 52, 58, 6, and 11) HPV vaccination among Kenyan women 15 to 20 years of age (10). The implementation science component of the KEN SHE Study sought to generate insights into the acceptability of the single-dose schedule among health-care professionals in Kenya (28).

Here, we share findings from our experience applying the TFA in the context of HPV vaccination in Kenya to assess the relationship between different components of the framework, the reported acceptability of the single-dose schedule among clinicians, and predictors of acceptability among health-care professionals in Kenya.

### Study design, sample size calculation, and participants

A concurrent mixed-methods approach was used to capture comprehensive insights to describe the perspective of health-care professionals and assess the acceptability of the single-dose schedule. A concurrent mixed-methods design prioritizes qualitative and quantitative data equally, data on both components are collected concurrently and analyzed independently, and results are mixed to interpret findings (29,30). Targeted clinicians included nurses, clinical officers, pharmacists, pharmacy technicians, medical officers, and other relevant health-care professionals. A sample size calculation was conducted focusing on counties where KEN SHE study sites were based (Kiambu, Kisumu, and Nairobi City) (28). Assuming that each health facility had at least 1 health-care professional responsible for HPV vaccination, with a total of 1564 health facilities in the 3 KEN SHE counties (364 in Kiambu, approximately 1000 in Nairobi, and approximately 200 in Kisumu), we estimated the total number of health-care professionals responsible for HPV vaccination to be approximately 1564. Thus, we estimated that responses were needed from at least 309 clinicians to be able to assess the acceptability of the single-dose schedule among health-care professionals with a 95% confidence interval (CI) that the real value is ±5% of the survey results.

### Implementation science framework and selection of appropriate constructs

To assess the acceptability of the single-dose HPV vaccination among health-care professionals, we used the TFA to capture clinicians’ insights into the acceptability of the single-dose schedule as well as other key aspects of acceptability relevant to single-dose HPV vaccination. To achieve this end, we reviewed the TFA questionnaire to select and adapt questions that aligned with constructs and domains relevant to our topic and context (ie, single-dose HPV vaccination in Kenya) (23). Additional information about this process is presented in the supplementary materials (Supplementary Methods 1, available online).

### Data collection and key study measures

Data were collected using a Research Electronic Data Capture (REDCap) survey developed based on domains of the TFA relevant to the context of HPV vaccination in Kenya (Supplementary Methods 2, available online) (22,23,31). Survey responses included qualitative data from free-text responses and quantitative data on participants’ demographic characteristics, their perspectives on the acceptability of the single-dose schedule, and relevant TFA domains. The survey was validated by 6 health-care professionals and other KEN SHE research team members before being deployed. Given the structure of the health-care system in Kenya that has 6 levels, from the community facilities (level 1) to national referral hospitals (level 6), we added an indicator to capture the level of the facility of respondents (32,33). The survey was shared with health-care professionals through WhatsApp to facilitate the wide distribution of the survey among clinicians involved in HPV vaccine delivery at different levels of the health-care system (urban vs rural, level 1 vs higher levels, etc). Additional details on the study design and procedures has been described elsewhere (28).

### Analytic approach

Descriptive statics were generated to describe demographic characteristics of survey respondents as well as the distribution of response across counties. We used χ^2^ tests for continuous variables and Kruskal-Wallis tests for categorical variables to assess the distribution of survey responses between the targeted counties. The distribution of key TFA items and the general acceptability were reported across the 3 counties and among all participants. Following previously published guidance on the analysis of TFA items; correlation analyses were performed to assess the relationship between 1) the reported general acceptability and scores of TFA items (relevant constructs of acceptability) and 2) the reported general acceptability and the total mean score of relevant TFA items (relevant constructs of acceptability) (23). Spearman correlation coefficients and *P* values were reported. A multivariate regression analysis was conducted to assess the association between key TFA items and the reported general acceptability score.

Further, regression analyses were performed to describe factors associated with acceptability while accounting for confounding characteristics. The outcome of interest (reported general acceptability) was treated as a continuous variable. Factors associated with the acceptability of the single-dose schedule that were examined included the county, geographic location of the facility (rural vs urban), level of the facility within the health system, job title (nurse vs pharmacist vs clinical officer vs medical doctor vs other). In addition, we adjusted for confounding characteristics, including the age, sex, religion, and duration at their job. Model selection analyses were run to assess the model assumptions and identify the best-fitting model. The generalized linear model with log-link function and family “Poisson” was selected as the best-fitting model. Incidence rate ratios for the best-fitting model and 95% CIs were reported. Quantitative analyses were performed in R, version 4.3.1 (R Foundation for Statistical Computing, Vienna, Austria).

For free-text responses (qualitative data), a rapid qualitative approach was used to identify themes that emerged from text responses (34,35). An inductive approach was used by identifying codes emerging from responses, and a deductive approach was used by identifying codes from the responses that were related to the TFA (22). Codes were merged into broader themes that were in turn paired with findings from the quantitative analysis to provide a comprehensive assessment of what clinicians thought about the single-dose schedule, how they learned about the single-dose schedule, and factors that enable or impede their acceptability of a single-dose schedule of the HPV vaccination. Findings were presented using a joint display approach.

### Ethics

This study was approved by the Kenya Medical Research Institute’s Scientific Ethics Review Unit, Massachusetts General Hospital, and the University of Washington institutional review boards. All participants underwent an informed consent process before completing the survey.

## Results

### Respondent characteristics

**Figure 1.**
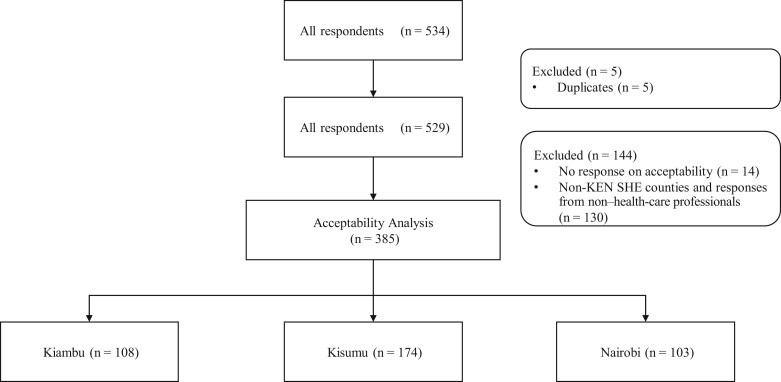
Flow chart describing responses used to assess the acceptability of the single-dose schedule of human papillomavirus vaccination for adolescent girls and young women among health-care professionals.

Overall, 534 responses from 35 counties were submitted to the REDCap survey. Because the survey was shared by Kenyan health-care professionals in different WhatsApp groups, we could not estimate the response rate because we did not know the number of participants in these groups. In total, 385 responses were included in the analysis after excluding duplicates (n = 5), entries that did not have an answer for the general acceptability question (n = 14), responses from nonclinician respondents (drivers, data clerks, etc), and non–KEN SHE counties (n = 130) (Figure 1). Among the 385 health-care professionals, 285 (74.0%) were women: 78.7% in Kiambu, 69% in Kisumu, and 77.7% in Nairobi (Table 1). The median age was 32 years overall, but the age was lower in Nairobi (28 years) (*P* = .004). Most respondents were Christian (97.1%), and 87.8% used a phone to complete the survey. The distribution of these characteristics was also consistent across counties.

**Table 1.** Demographic characteristics of respondents, 385 health-care professionals from the 3 counties

	**Kiambu** (n = 108)	**Kisumu** (n = 174)	**Nairobi** (n = 103)	**Overall** (n = 385)	*P* ^a^
Sex, n (%)					.097^b^
Male	22 (20.4)	54 (31)	23 (22.3)	99 (25.8)	
Female	85 (78.7)	120 (69)	80 (77.7)	285 (74.2)	
Age, median (IQR)	32.0 (12.5)	32.5 (9.0)	28.0 (8.0)	32 (10)	.004^c^^,^*
Religion, n (%)					.38^b^
Christian	107 (99.1)	165 (94.8)	101 (98.1)	374 (97.1)	
Muslim	1 (0.9)	6 (3.4)	1 (1.0)	8 (2.1)	
Other	0 (0)	2 (1.1)	1 (1.0)	3 (0.8)	
Modality, n (%)					.29^b^
In-person	7 (6.5)	11 (6.3)	3 (2.9)	21 (5.5)	
Phone	94 (87.0)	155 (89.1)	89 (86.4)	338 (87.8)	
Computer/laptop	5 (4.6)	8 (4.6)	10 (9.7)	23 (6.0)	
Role/cadre, n (%)					.029^b^
Nurse	50 (46.3)	76 (43.7)	61 (59.2)	187 (48.6)	
Clinical officer	21 (19.4)	24 (13.8)	8 (7.8)	53 (13.8)	
Medical officer	4 (3.7)	7 (4.0)	10 (9.7)	21 (5.5)	
Pharmacy technologist	3 (2.8)	10 (5.7)	5 (4.9)	18 (4.7)	
Pharmacist	2 (1.9)	4 (2.3)	3 (2.9)	9 (2.3)	
Hospital director	2 (1.9)	1 (0.6)	0 (0)	3 (0.8)	
Other	25 (23.1)	51 (29.3)	16 (15.5)	92 (24.2)	
Duration at the job, median (IQR), months	48.0 (72)	60.0 (72)	36.0 (60)	60.0 (72)	.052^c^^,^*
Level in the health-care system of the facility, n (%)					<.05^b^^,^*
Community facility (level 1)	4 (3.7)	3 (1.7)	4 (3.9)	11 (2.9)	
Health dispensary (level 2)	12 (11.1)	8 (4.6)	0 (0)	20 (5.2)	
Health center (level 3)	14 (13.0)	37 (21.3)	23 (22.3)	74 (19.2)	
County hospital (level 4)	20 (18.5)	84 (48.3)	24 (23.3)	128 (33.2)	
County referral (level 5)	31 (28.7)	30 (17.2)	17 (16.5)	78 (20.3)	
National referral (level 6)	7 (6.5)	1 (0.6)	15 (14.6)	23 (6.0)	
Other	20 (18.5)	11 (6.3)	18 (17.5)	49 (12.7)	
Location of the health facility, n (%)					<.05^b^^,^*
Urban	85 (78.7)	112 (64.4)	95 (92.2)	292 (75.8)	
Rural	23 (21.3)	61 (35.1)	5 (4.9)	89 (23.1)	
Other	0 (0)	0 (0)	2 (1.9)	2 (0.5)	

a *P* value comparing characteristics across KEN SHE counties.

b Kruskal-Wallis test.

c χ^2^ test.

* *P* <.05.

Overall, most respondents were nurses (48.6%) or had other job titles (24.2%), such as community health-care workers, community health volunteers, and specialists such as pediatricians and gynecologists. Other job titles included clinical officers (13.8%), medical officers (5.5%), and pharmacists (4.7%). Respondents had been in their job for an average of 60 months, with a variation across counties, and the lowest average duration of 36 months (approximately 3 years) was reported among respondents from Nairobi. In addition, referral levels where respondents were employed included level-4 facilities (33.2%), level-5 facilities (20.3%), and level-3 facilities (19.2%). This distribution differed across sites; in Kiambu, 11.1% of respondents were based at health dispensaries (level 2), and in Nairobi, 14.6% were employed at level-6 facilities (*P* < .05). Most respondents’ facilities were in urban settings (75.8%), and this proportion varied across counties (92.2% in Nairobi, 78.7% in Kiambu, and 64.4% in Kisumu) (Table 1).

### Acceptability constructs and operationalization of the TFA

Overall, most health-care professionals reported that a single-dose HPV vaccination schedule for adolescent girls and young women was acceptable (43.38%) or very acceptable (32.46%). Regarding domains of the TFA, in terms of burden, most clinicians thought that a single-dose schedule did not require a substantial effort (69.87%), a large amount of time (87.02%), or considerable resources (77.41%). For ethics, 32.38% of health-care professionals agreed or completely agreed that there were moral consequences to offering a single-dose schedule to adolescent girls and young women, while 83.07% reported that giving a single-dose of HPV vaccination to adolescent girls and young women was fair (Table 2). Most health-care professionals (89.29%) reported that the single-dose HPV vaccination schedule was effective in preventing cervical cancer, 86.94% were confident that providing a single-dose schedule to adolescent girls and young women, while 23.75% of health-care professionals reported not having knowledge of the single-dose schedule for HPV vaccination. The distribution of responses on the general acceptability portion of the TFA and relevant aspects of acceptability from the TFA were similar across the 3 counties (Supplementary Figures 1, A-F, available online). Overall, free-text responses highlighted that health-care professionals thought that the single-dose schedule was convenient, safe, effective, resource friendly, and would result in increased uptake of HPV vaccination. Parental acceptability was reported as a major concern, or *“the elephant in the house”* (Table 2). Some health-care professionals were misinformed since they thought that the HPV vaccine causes cancer and infertility and should not be given to adolescent girls and young women. Other concerns reported related to the acceptability of the single-dose schedule included clinicians’ knowledge of the matter and ability to address concerns from adolescent girls and young women or their parents. In addition, in terms of other key insights captured, 78.12% of health-care professionals also reported that integrating the single-dose schedule within the existing workflow would not require huge effort, 89.52% were confident explaining benefits of HPV vaccination, and 91.91% were confident addressing concerns about HPV vaccination from adolescent girls and young women and their parents or guardians (Table 3). Free-text responses underscored the gap in communication and sensitization for HPV vaccination. This issue was raised in responses from health-care professionals, who mentioned that “There was no proper mobilization about HPV vaccines,” “They need more information and sensitization to the staff and parents,” and they “need [to] be able to explain the importance of vaccine to the guardian in local, understandable language.” They flagged that more training would equip them with appropriate skills (Table 3). Finally, additional insight highlighted that despite many challenges that they thought might impede their ability to deliver the single-dose schedule, most health-care professionals considered the single-dose schedule to be “a good thing to be implemented” and the “way forward.”

**Table 2.** Responses of participants on the acceptability of a single-dose HPV vaccination schedule for adolescent girls and young women (N = 385)

Domain	Subdomain	**Question** ^a^	Completely disagree, n (%)	Disagree, n (%)	Neutral, n (%)	Agree, n (%)	Completely agree,n (%)	Insights from health-care professionals
**General Acceptability**	—	Is a single-dose of HPV vaccination for adolescent girls and young women acceptable?	52 (13.51)	9 (2.34)	32 (8.31)	167 (43.38)	125 (32.46)	Believed that a single-dose schedule would be more acceptable than the 2-dose schedule Noted that it would result into increased uptake and coverage Emphasized acceptability (among health-care professionals and the community) as a factor that would enable their ability to deliver a single-dose schedule Acceptability among parents and caregivers reported as the major concern, or “the elephant in the house.”
**Burden** ^b^	Effort	Is a huge effort needed to provide single-dose HPV vaccination?	52 (13.51)	217 (56.36)	63 (16.36)	37 (9.61)	16 (4.16)	Noted that a single-dose schedule would require “minimal effort” and result in better compliance Regarded the single-dose schedule as a good approach that was “efficient and less tedious and that would reduce workload” Flagged that a single-dose schedule would “reduce contact tracing or follow-up efforts that have been challenging in the past”
	Time	Is a huge amount of time needed to provide single-dose HPV vaccination?	11 (2.86)	324 (84.16)	25 (6.49)	22 (5.71)	3 (0.78)	Stressed the efficiency and reduced time consumption of the single-dose schedule as a factor that would facilitate its delivery Pointed out that using less time to deliver the single-dose schedule would increase its acceptability among health-care professionals: “[I]t saves time and the turn out will be good.” Considered a single-dose schedule “very easy and saves time since there is no need to come back.”
	Resource	Are huge resources needed to provide single-dose HPV vaccination?	14 (3.64)	284 (73.77)	26 (6.75)	47 (12.21)	14 (3.64)	Reported the lack of required resources (cold chain, consumables, storage, transport, communication materials, adequate workforce) as a factor limiting their confidence in delivering the single-dose schedule Mentioned that a single-dose schedule will reduce financial resources needed and the rate of defaulters Believed that a single-dose schedule was “resource friendly.” Recommended that the single-dose schedule be “adopted especially in government facilities.”
**Ethicality**	Moral consequence	Are there moral consequences to offering single-dose HPV vaccination?	72 (18.80)	113 (29.50)	74 (19.32)	98 (25.59)	26 (6.79)	Reported concerns of people who thought that the vaccine causes infertility and others who thought that it causes cancer. “It can cause cancer.”
	Fairness	Is it fair to give a single-dose HPV vaccination to adolescent girls and young women?	19 (4.95)	15 (3.91)	31 (8.07)	147 (38.28)	172 (44.79)	Believed that a single-dose schedule will be beneficial to adolescent girls and young women: *“*It will offer many girls protection because not all girls receive a second dose.”
**Perceived Effectiveness**	Effectiveness of 1 dose	Is a single-dose HPV vaccination effective in preventing cervical cancer?	5 (1.31)	23 (6.0)	13 (3.39)	157 (40.99)	185 (48.30)	Many reported that a single-dose prevents HPV infection and cervical cancer: “No significant different in its efficacy compared to multiple doses.” Single-dose for live immunity” Reported that it might give partial protection Uncertainty in its effectiveness was also reported by some health-care professionals: “Not sure if it is as effective as 2 dose” “It’s working but not sure of its effectiveness”
**Self-Efficacy**	—	Are you confident providing single-dose of HPV vaccination?	22 (5.74)	12 (3.13)	16 (4.18)	91 (23.76)	242 (63.18)	Reported confidence as an enabling factor in delivering a single-dose schedule Most health-care professionals reported being confident delivering the single-dose schedule Some mentioned that awareness will need to be increased properly to preserve their confidence: “If awareness creation will not be done properly, then it might interfere with my confidence”
**Intervention Coherence**	Knowledge of the single-dose schedule	Are you knowledgeable about the single-dose schedule for HPV vaccination?	34 (8.97)	56 (14.78)	25 (6.60)	190 (50.13)	74 (19.52)	Reported minimal or little knowledge of the single-dose schedule Cited knowledge, skills, and training gaps as major barriers to delivering a single-dose schedule: “Lack of knowledge on how to deal with adverse effects in case they occur” Noted that they knew details regarding single-dose schedule delivery (frequency, mode of delivery, administration, and the target population) Highlighted benefits (effectiveness, efficiency, potential to improve uptake and compliance)” “Given once without a second dose” “World Health Organization approved” “Same or simply efficacious to the double dose, better uptake”

a Questions were reworded for brevity; Supplementary Methods 2 (available online) presents questions that were asked to apply the TFA and capture constructs and domains of acceptability relevant to this project. HPV = human papillomavirus; TFA = Theoretical Framework of Acceptability.

b Constructs for the burden domain were reverse-scored during analysis to make sure that the high score/ranking corresponded to high acceptability (ie, if the evidence-based intervention requires a little effort, it is more likely to be acceptable).

**Table 3.** Other insights related to the acceptability of the single-dose schedule reported by health-care professionals (N = 385)

Domain/theme	Construct/subtheme	Completely disagree,n (%)	Disagree, n (%)	Neutral, n (%)	Agree, n (%)	Completely agree, n (%)	Insights from health-care professionals
**Burden** ^a^	Integration of single-dose vaccination into existing work	29 (7.55)	271 (70.57)	24 0(6.25)	50 (13.02)	10 (2.60)	Reported the lack of integration into the normal flow as an obstacle limiting their confidence to deliver the single-dose HPV vaccination schedule
**Perceived Effectiveness**	Effectiveness of the HPV vaccine	2 (0.52)	15 (3.91)	9 (2.34)	154 (40.10)	204 (53.12)	Voiced that the HPV vaccine was safe and effective Considered the effectiveness and efficacy of the vaccine as a factor that would facilitate the delivery of the single-dose schedule: “I have had the shot, I know of the advantages of the vaccine, I know it will greatly reduce cervical cancer in our population.”
**Self-Efficacy**	Confidence explaining benefits	10 (2.61)	18 (4.71)	12 (3.14)	79 (20.68)	263 (68.84)	Mentioned that more training would empower them to communicate well advantages of HPV vaccination to adolescent girls and young women, parents, and the community: “Need be able to explain the importance of vaccine to the guardian in local, understandable language”
	Confidence addressing concerns	10 (2.61)	10 (2.61)	11 (2.87)	109 (28.46)	243 (63.45)	Reported that capacity building would enhance their confidence in counselling and administering the HPV vaccine
**Additional insights reported**							
**Facilitators of single-dose delivery**	Availability of clear protocols, policy, and guidelines from the Ministry of Health Motivation (of adolescent girls and young women as well as health-care professionals) Proper planning Efficacy, effectiveness, and safety of the HPV vaccine Simplified school-based vaccination Sensitization, awareness, engagement, and health education of adolescent girls and young women, parents, and the community Health-care professionals’ skills (including clinical, outreach, and community engagement experience) Availability of training and mentorship						
**Barriers to single-dose delivery**	Vaccine hesitancy Religious and cultural beliefs Myths and misconceptions about the HPV vaccine Community norms and traditions Lack of information about or updates on the vaccine among health-care professionals and the community Lack of support from the Ministry of Health and parents Refusal and lack of consent from parents or adolescent girls and young women Lack of knowledge, awareness, mobilization of and sensitization to the single-dose schedule in the community Reported stigma as a concern that was still present; “stigma is still there”						
**Belief in single-dose schedule**	Considered the single-dose schedule “a good thing to be implemented” Reported concerns related to its protection or whether a boaster dose will be needed later: “Not very sure if the content is efficient to offer full protection compared to the recommended doses.” “I actually do not know if one will need a booster later”						
**Other comments**	Learned about the single-dose schedule mostly from colleagues, the internet, training and continuing medical education, or workshops Some reported that they had yet to learn about it: “I have not heard about it.” “I haven’t learnt as yet” “Not yet updated” “Learned through this survey, now”						

a Constructs for the burden domain were reverse-scored during analysis to make sure that the high score/ranking corresponded to high acceptability (ie, if the evidence-based intervention requires a little effort, it is more likely to be acceptable). HPV = human papillomavirus.

When assessing the relationship between the general acceptability measure and responses reported on relevant constructs of acceptability, all constructs had a weak or very weak correlation with the general acceptability measure. The resource and moral consequence constructs had a very weak negative correlation with the reported acceptability (Spearman correlation coefficient = ‒0.007 and ‒0.056, respectively), but these relationships were not statistically significant. In contrast, the fairness and effectiveness constructs had a positive correlation with acceptability (Spearman correlation coefficient = 0.24 and 0.14, respectively; *P* < .05) (Table 4). When assessing the relationship between the reported general acceptability and the acceptability measurement calculated by getting an average of relevant constructs, the new acceptability scores were weakly correlated with the reported general acceptability, and this relationship was statistically significant for the acceptability scores calculated, including fairness as 1 of the constructs (Table 5; Supplementary Table 1, available online). When assessing the association between the constructs of acceptability with the general acceptability measure, most constructs were found to be weak predictors of acceptability, except for the fairness construct (β = .23, 95% CI = 0.1 to 0.36; *P* < .001) (Supplementary Table 2, available online). Finally, all relevant constructs of acceptability remained weakly correlated with the general acceptability measure when these variables were treated as categorical variables (Supplementary Table 3, available online).

**Table 4.** Correlation between the acceptability domains and general acceptability reported

Domain	Constructs	Spearman correlation	
		Coefficient	*P*
**Burden** ^a^	Effort to provide single-dose	0.0011	.98
	Time to provide single-dose	0.028	.58
	Resource to provide single-dose	−0.007	.89
**Ethicality**	Moral consequence to providing single-dose	−0.056	.27
	Fairness to provide single-dose	0.24	<.01^b^
**Self-Efficacy**	Confidence	0.069	.18
**Perceived Effectiveness**	Effectiveness of single-dose	0.14	<.05^b^
**Intervention Coherence**	Knowledge on single-dose	0.034	.51
**Other insights captured that aligned with domains of acceptability**			
**Burden** ^a^	Effort required to integrate the HPV vaccine in an existing workflow	0.013	.8
**Self-Efficacy**	Confidence explaining benefits of the vaccine	0.059	.25
	Confidence addressing concerns on the vaccine	0.05	.33
**Perceived Effectiveness**	Effectiveness of the HPV vaccine	0.12	<.05^b^

a Constructs for the burden domain were reverse-scored to make sure that the high score/ranking corresponded to high acceptability (ie, if the evidence-based intervention requires a little effort, it is more likely to be acceptable). HPV = human papillomavirus.

b *P* <.05 (these constructs and general acceptability are significantly correlated with low positive correlation coefficient and *P* < .05).

**Table 5.** Correlation between the reported general acceptability and the acceptability computed from relevant domains^a^

		Distribution of score across counties, mean (SD)^b^					Spearman correlation	
Item	Component	Kiambu	Kisumu	Nairobi	Overall	*P* ^c^	Coefficient	*P* ^d^
General acceptability (reported)	NA	4.01 (1.30)	3.74 (1.33)	3.65 (1.22)	3.79 (1.29)	.1	—	—
Acceptability mean score1^e^	Effort Moral consequence Confidence Effectiveness Knowledge	3.76 (0.51)	3.71 (0.52)	3.70 (0.51)	3.72 (0.52)	.69	0.052	.31
Acceptability mean score2^f^	Effort Fairness Confidence Effectiveness Knowledge	4.10 (0.59)	3.98 (0.62)	3.95 (0.51)	4.0 (0.58)	.15	0.18	<.05^*^

a Full table available in the Supplementary Materials (Supplementary Table 1, available online). NA = not applicable.

b Mean acceptability scores were calculated by summing the score for relevant constructs for each participant and dividing the total score by the number of constructs used for the calculation (5).

c *P* value comparing scores across sites.

d *P* value of the correlation coefficient.

e Using moral consequence as the measurement of burden, the newly computed acceptability score was very weakly positively correlated with the general acceptability score reported (ρ = 0.052). This correlation was not statistically significant (*P* = .3).

f Using fairness to assess burden, the newly calculated acceptability score was weakly positively correlated with the general acceptability score reported (ρ = 0.18). This relationship was statistically significant (*P* < .05).

* *P*-value < .05.

### Model selection

Although the outputs from the multivariate logistics regression showed that no statistically significant association between characteristics of health-care professionals and the acceptability of the single-dose schedule, the linear multivariate model showed that other cadres had 0.37 higher acceptability (as a unit of the acceptability score) than nurses (95% CI = 0.01 to 0.73; *P* = .044) (Supplementary Table 4, available online). When assessing the combined effect of the referral level and county as well as the job title and duration on the acceptability of the single-dose schedule, the referral level had a linear, quadratic, and cubic effect on the acceptability of the single-dose schedule, where this effect resulted in increased acceptability across referral levels in the linear and cubic trend (Supplementary Table 5, available online). In addition, outputs from this model revealed that health-care professionals from Nairobi had 0.43 lower acceptability of the single-dose schedule than those in Kiambu (95% CI = ‒0.84 to ‒0.02; *P* = .039).

To assess the linearity assumption of the multivariate linear regression model with and without interactions, we assessed the distribution of residuals compared to predicted values and found that residuals seemed to follow a pattern and were not random in nature. Thus, a logistic regression model was more appropriate for our data (Supplementary Table 6, available online). In addition, the Akaike information criterion (AIC) revealed that the multivariate regression without interaction was indeed the best-fitting model and most appropriate for our data (Supplementary Figure 2, available online). Finally, we assessed the possible effect of fairness on the acceptability of a single-dose schedule and found that although an increased score for fairness was statistically significantly associated with greater acceptability of the single-dose schedule among health-care professionals, this variable had influence on the effect of clinicians’ characteristics on the acceptability of the single-dose schedule (Supplementary Figure 3, available online). Based on these results, we reported findings from the simplest logistic regression (no interaction or constructs of acceptability added) to estimate the association between health-care professional characteristics and the acceptability of the single-dose HPV vaccination schedule.

### Determinants of acceptability

**Figure 2.**
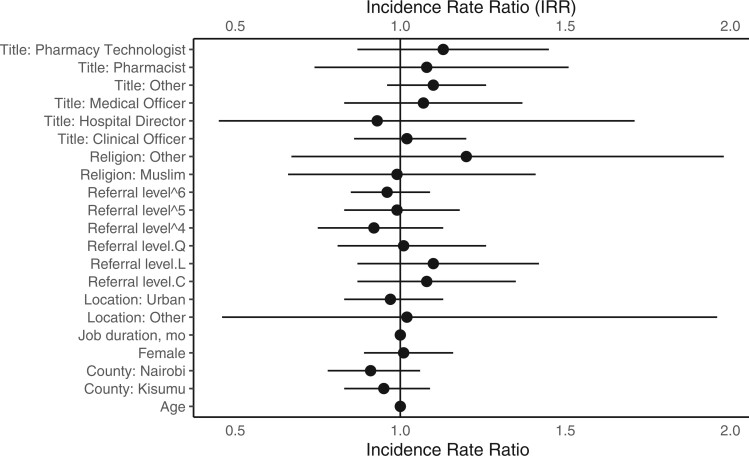
Determinants of acceptability among health-care professionals. Findings of a multivariate Poisson regression reporting incidence rate ratios with 95% confidence intervals. Model equation: logit (Y_m_) = α_m_ + B1 job title + B2 religion + B3 referral level + B4 geographic location of the facility + B5 job duration + B6 gender + B7 county + B8 age.

When assessing the association between health-care professional characteristics and their acceptability of the single-dose schedule, while controlling for other factors we found that the acceptability was 1% higher among female health-care professionals than among male professionals (incident rate ratio = 1.01, 95% CI = 0.89 to 1.16) and 20% higher among clinicians of other religions than among Christians (incident rate ratio = 1.2, 95% CI = 0.67 to 1.98) (Table 5). In addition, acceptability of the single-dose schedule was 13% higher among pharmacy technologist (incident rate ratio = 1.13, 95% CI = 0.87 to 1.45), 10% higher among other cadres (incident rate ratio = 1.1, 95% CI = 0.96 to 1.26), but 7% lower among hospital directors (incident rate ratio = 0.93, 95% CI = 0.45 to 1.71) compared to nurses. In terms of the locations of their facilities, the acceptability of the single-dose schedule was 3% lower among health-care professionals based in urban settings than among clinicians in rural areas (incident rate ratio = 0.97, 95% CI = 0.83 to 1.13); acceptability of the single-dose schedule among health-care professionals in Kisumu and Nairobi was lower by a factor of 0.95 (incident rate ratio = 0.95, 95% CI = 0.83 to 1.09) and 0.91 (incident rate ratio = 0.91, 95% CI = 0.78 to 1.06), respectively, compared with Kiambu, while holding other clinicians’ characteristics constant (Table 6 and Figure 2). Finally, no contrasts contributed significantly to explaining the difference between referral levels and the acceptability of the single-dose schedule. We found a linear increasing trend in acceptability across referral levels, but there was no significant effect of the referral level on the acceptability of the single-dose schedule (incident rate ratio = 1.1, 95% CI = 0.87 to 1.42). A similar pattern was observed for the quadratic and cubic trend, but higher-level trends were associated with a decreased trend in acceptability across referral levels. Overall, the referral level did not have a linear, quadratic, or cubic effect on the acceptability of the single-dose schedule among health-care professionals.

**Table 6.** Determinants of acceptability among health-care professionals, with findings of the multivariate Poisson regression reporting incidence rate ratios with 95% confidence intervals

	Incident rate ratio	95% Confidence interval	*P*
Sex			
Male	[Reference]	[Reference]	
Female	1.01	0.89 to 1.16	.8
Age	1	0.99 to 1.01	.6
Religion			
Christian	[Reference]	[Reference]	
Muslim	0.99	0.66 to 1.41	>.9
Other	1.2	0.67 to 1.98	.5
Job title			
Nurse	[Reference]	[Reference]	
Clinical officer	1.02	0.86 to 1.20	.8
Medical officer	1.07	0.83 to 1.37	.6
Pharmacy technologist	1.13	0.87 to 1.45	.3
Pharmacist	1.08	0.74 to 1.51	.7
Hospital director	0.93	0.45 to 1.71	.8
Other	1.1	0.96 to 1.26	.2
Job duration, mo	1	1.00 to 1.00	.8
Facility level			
Facility_level.L	1.1	0.87 to 1.42	.4
Facility_level.Q	1.01	0.81 to 1.26	>.9
Facility_level.C	1.08	0.87 to 1.35	.5
Facility_level^4	0.92	0.75 to 1.13	.4
Facility_level^5	0.99	0.83 to 1.18	.9
Facility_level^6	0.96	0.85 to 1.09	.5
Geographic location			
Rural	[Reference]	[Reference]	
Urban	0.97	0.83 to 1.13	.7
Other	1.02	0.46 to 1.96	>.9
County			
Kiambu	[Reference]	[Reference]	
Kisumu	0.95	0.83 to 1.09	.5
Nairobi City	0.91	0.78 to 1.06	.2

## Discussion

In this study, we applied the TFA within the context of HPV vaccination to assess the acceptability of a single-dose schedule among health-care professionals across 3 Kenyan counties (Kiambu, Kisumu, and Nairobi). Overall, most clinicians (75.84%) reported that a single-dose HPV vaccination schedule for adolescent girls and young women was acceptable or very acceptable. Most respondents were women and nurses and had been in their job for an average of 60 months. Thirty-three percent were based at level-4 facilities, and 75.8% were in urban settings. Close to one-third of health-care professionals were not knowledgeable about the single-dose vaccination schedule, while 93% of clinicians thought that HPV vaccination was effective in preventing cervical cancer and a slightly lower proportion (89.3%) thought that a single-dose schedule was effective in preventing cervical cancer. This finding was emphasized by insights reported qualitatively by health-care professionals who mentioned that a single-dose HPV vaccination schedule was efficacious in preventing or protecting against cervical cancer. These findings converged with quantitative results and provided more insights into health-care professionals’ knowledge of and beliefs about the single-dose schedule. Focusing on clinician characteristics, we found that hospital directors had 7% lower acceptability than nurses, while pharmacists and other cadres had 13% and 10%, respectively, higher acceptability of the single-dose schedule than nurses. In addition, health-care professionals based in urban settings had lower acceptability than those in rural settings. Constructs of the TFA were weakly correlated to and were weak predictors of the general measure of acceptability, which have practical and theoretical implications.

Practically, from a public health perspective, we identified areas to improve HPV vaccination uptake in Kenya. In a context where health-care professionals are considered a main source of information when making health decisions, it is important to ensure that they are equipped with the latest evidence on cervical cancer prevention and control to accelerate uptake of HPV vaccination. Other studies previously reported that women obtained information about HPV vaccine from health-care professionals (20,36). Similar results were reported in Cameroon, where a study conducted among 553 adolescent girls reported that 62.9% heard about HPV from a nurse. This value was higher than from other sources, such as teachers (14.2%) or the public media (13.5%) (37). Irrespective of HPV vaccination schedule (1 vs 2 doses), in our study, some health-care professionals reported that the HPV vaccine was not effective in preventing cervical cancer. This opinion is concerning because a recent systematic review of health-care professionals’ HPV vaccine perceptions, hesitancy, and recommendation to patients highlighted that their recommendation had a considerable impact on patient uptake across vaccines and populations. In addition, clinicians’ confidence in and knowledge of the vaccine, disease severity, and risk of infection were associated with a high likelihood to recommend the vaccine. Similar findings were reported in other settings, such as in Cameroon, where 44% of health-care professionals reported not believing that the HPV vaccine prevented cancer but a high willingness to recommend the vaccine was reported among clinicians who believed that it could prevent cancer (19). Health-care professionals play an important role in the promotion and delivery of immunization and preventive services in many sub-Saharan African settings, and they are trusted sources of information in their communities; thus, they can contribute substantially to increasing uptake.

Theoretically, although the TFA has been used in other contexts, most studies have used it to assess acceptability qualitatively (38-41). Our use of the TFA to capture and assess acceptability quantitative highlighted that constructs of the TFA that were thought to capture aspects of acceptability may not be facets or constituents of acceptability. This finding raises a question about the TFA and suggests a need to reassess whether respondents’ understanding of key components of acceptability align with what was proposed during the design and validation of the TFA. As previously reported, the TFA was developed with a focus on insights from biomedical studies that were conducted in high-income countries, which may affect its relevance or appropriateness for LMICs (22). In addition, the TFA was designed with input from researchers from high-income countries, with low representation from nonacademic stakeholders which might have excluded important considerations from recipients of evidence-based interventions and perspectives from LMICs. Although other theorical frameworks have been developed that expand on the TFA, it may be worth assessing the perspective of stakeholders beyond the research community on the relevance of constructs and domains that have been portrayed as key aspects or components of acceptability (25,26,42).

Considering vaccination schedule, our study highlighted that more than one-third of the respondents were not knowledgeable about the single-dose schedule, and a similar proportion thought that there were moral consequences to offering the single-dose schedule to adolescent girls and young women. Although results from the logistic regression were not statistically significant, they should be considered given their public health relevance, which could contribute to increasing HPV vaccination uptake in these settings. If Kenya considers a single-dose schedule to accelerate progress on cervical cancer prevention, it will be crucial to use appropriate approaches to increase knowledge on single-dose vaccination among health-care professionals, address concerns they may have about moral consequences of a single-dose schedule, and engage key stakeholders such as hospital directors to get their buy-in and improve their acceptability of the single-dose schedule before roll-out. Other considerations for a successful implementation should include using appropriate strategies for health-care professionals based in urban settings as well as strategies that account for a county-specific implementation context. In addition to equipping health-care professionals with the right educational materials, communication training may also have an impact, making sure that health-care professionals appropriately address the concerns of parents, adolescent girls and young women, and the community regarding HPV vaccines that would increase uptake (21). We captured insights into acceptability among health-care professionals, but to ultimately achieve HPV vaccination and cervical cancer elimination goals, the perspective of 2 other key elements—their acceptability of the single-dose schedule and other key contextual characteristics that could affect HPV vaccination delivery—should be considered (43).

One limitation of this study is that the health-care professionals who participated in the study may not be representative of all health-care professionals involved in HPV vaccination. To capture the most representative sample possible, we aimed to use health-care professional WhatsApp groups to survey clinicians from different levels of the health-care system; we may also have been able to capture insights from clinicians not on WhatsApp groups. In addition, because contact information for all health-care professionals involved in HPV vaccination in the 3 counties was not widely available, we could not estimate the response rate. Furthermore, our findings might have not explored some aspects in depth, which, for example, could have included a further exploration of why some health-care professionals thought that there was a moral consequence to providing a single-dose HPV vaccination schedule to adolescent girls and young women while a considerable proportion of health-care professionals thought that a single-dose HPV vaccination schedule for this group was fair. Despite these limitations, our findings generated early insights into health-care professionals’ perspective regarding the single-dose schedule in Kenya. Our findings also provided insights into the application of the TFA to capture data and assess acceptability quantitatively in the context of HPV vaccination in an LMIC. In addition, these findings could provide useful insights into the National Vaccine and Immunization Program and contribute to the design and adaptation of approaches to improve HPV vaccine uptake in Kenya.

Pairing tailored educational approaches for key stakeholders and removing the limitation of a multiple-vaccination schedule could increase acceptability. A single-dose schedule has the potential to address concerns related to financial burden and access, which would also improve acceptability and ultimately contribute to increasing uptake. Given the reduced logistics needed to deliver a single-dose schedule, this approach has the potential to catalyze equitable access to HPV vaccination by reaching adolescent girls and young women who may be in complicated situations due to their community livelihood, such as nomad communities, or who are in remote or conflict areas.

## Supplementary Material

lgae031_Supplementary_Data

## Data Availability

The raw data underlying this article cannot be shared because of ethical reasons to protect the identity of individuals who participated in the study. Facility-level demographics can be shared as well as individual-level data, excluding information that could trace the responses back to study participants, especially in small facilities. The data will be available upon request through on an online secure repository.
