# Supplementary material for: Acceptability of single-dose HPV vaccination schedule among health-care professionals in Kenya: a mixed-methods study
Source: J Natl Cancer Inst Monogr. 2024 Nov 12;2024(67):358–70. doi: 10.1093/jncimonographs/lgae031 (PMC11555271; doi:10.1093/jncimonographs/lgae031)

Supplementary Materials

Supplementary Methods

- **Supplementary Methods 1:** Approach used to select relevant TFA constructs and adapt the TFA questionnaire
- **Supplementary Methods 2:** Data collection Tool

Supplementary Tables

- **Supplementary Table 1:** Correlation between the reported general acceptability and the computed acceptability varying constructs used to represent relevant domains (with all variables treated as continuous variable)
- **Supplementary Table 2:** Output from the regression model assessing the association between constructs of acceptability and the reported general acceptability.
- **Supplementary Table 3:** Correlation between the general acceptability and the different constructs of acceptability (Treated as categorical variables)
- **Supplementary Table 4:** Determinants of acceptability among HCPs. Findings of a multivariate linear regresion model (without interraction)
- **Supplementary Table 5:** Determinants of acceptability among HCPs. Findings of a multivariate linear regresion model (with interraction between referral level and county as well as job title and job duration)
- **Supplementary Table 6:** Model selection: Checking the best fitting model

Supplementary Figures

- **Supplementary Figures 1:** Distribution of responses on domains of acceptability overall and across counties
- **Supplementary Figure 2:** Model selection: Assessing the linearity assumption for the linear regression model with lowest AIC values**Supplementary Figure 3:** Comparison of estimates of determinants of acceptability among HCPs. Findings of a multivariate logistic regression with and without the fairness construct included in the model, reporting incidence rate ratios (IRR) with 95% Confidence Intervals (CI)
- **Supplementary Figure 4:** Determinants of acceptability among HCPs. Findings of a multivariate poisson regresion reporting incidence rate ratios (IRR) with robust standard errors (HC1)

Supplementary Methods

Supplementary Methods 1: Approach used to select relevant TFA constructs and adapt the TFA questionnaire

The theoretical framework for acceptability (TFA) has 7 components constructs as described by Sekhon and colleagues.[22,23] The TFA was leveraged to inform the design of the survey used in our study by focusing on questions that were relevant to the context of single-dose HPV vaccination in our setting. All constructs were included in our questionnaire except for the construct of “affective attitude” and “opportunity costs” since they were not deemed relevant to our context. Additional questions were added to capture other aspects of burden (time, effort or resources) that were crucial from HCPs’ perspective to deliver a single-dose HPV vaccination. With input from local implementers, we adjusted the construct of intervention coherence to capture HCPs’ knowledge of the single-dose HPV vaccination schedule. Additionally, with feedback from local partners, additional questions were added to the construct of “perceived effectiveness” to explore HCPs’ perceived effectiveness of the HPV vaccine in preventing HPV infections in addtion to capturing their perceived effectiveness of the single-dose. Questions were also added to the construct of self-efficacy to assess HCPs’ confidence in addressing concerns and explaining benefit of the HPV vaccine to parents and AGYW in addition to assesssing their confidence in providing the single-dose HPV vaccine to AGYW. Selected constructs and related questions were validated and pilot tested with HCPs in Kenya before launching the survey. Quantitative data from survey responses were analyzed following the guidance from Sekhon and colleagues while qualitative data from free text responses were analyzed using a rapid qualitative analysis approach.[23,34,35] The table below summarizes the 7 TFA constructs, their definition, the generic questionnaire items as well as related quantitative questions that were included in our adapted TFA questionnaire. A copy of the survey with all the questions that were asked HCPs is available in the Supplementary Methods 2.

<table>
<tr><th>TFA construct</th><th>Definition</th><th>Generic TFA questionnaire items</th><th>Questionnaire items from the survey</th></tr>
<tr><td>Affective attitude</td><td>How an individual feels about the intervention</td><td>Did you like or dislike [intervention]?
OR
How comfortable did you feel [behaviour i.e. to engage with] [intervention]?</td><td>NA</td></tr>
<tr><td>Burden</td><td>The amount of effort required to participate in the intervention</td><td>How much effort did it take [behaviour i.e. to engage with] [intervention]?
<table>
<tr><td>No effort at all</td><td>A little effort</td><td>No opinion</td><td>A lot of effort</td><td>Huge effort</td></tr>
<tr><td>1</td><td>2</td><td>3</td><td>4</td><td>5</td></tr>
</table></td><td>How much effort does it take you to provide one dose of the HPV vaccine to AGYW?[a]
<table>
<tr><td>No effort at all</td><td>A little effort</td><td>No opinion</td><td>A lot of effort</td><td>Huge effort</td></tr>
<tr><td>1</td><td>2</td><td>3</td><td>4</td><td>5</td></tr>
</table>
In your opinion, how much time does it/ will it take you to provide a single-dose of the HPV vaccine to AGYW?[a]
<table>
<tr><td>No time at all</td><td>Little time</td><td>No opinion</td><td>A lot of time</td><td>Huge amount of time</td></tr>
<tr><td>1</td><td>2</td><td>3</td><td>4</td><td>5</td></tr>
</table>
In your opinion, how much resource does it/ will</td></tr>
</table>

<table>
<tr>
<td></td>
<td></td>
<td></td>
<td>it take you to provide a single-dose of the HPV vaccine to AGYW?[a]
<table>
<tr><td>No resource at all</td><td>Little resource</td><td>No opinion</td><td>A lot of resources</td><td>Huge resources</td></tr>
<tr><td>1</td><td>2</td><td>3</td><td>4</td><td>5</td></tr>
</table>
. How much effort does it take to incorporate the Single-dose into the existing workflow at your clinic/ facility?[b]
<table>
<tr><td>No effort at all</td><td>A little effort</td><td>No opinion</td><td>A lot of effort</td><td>Huge effort</td></tr>
<tr><td>1</td><td>2</td><td>3</td><td>4</td><td>5</td></tr>
</table>
</td>
</tr>
<tr>
<td>Ethicality</td>
<td>The extent to which the intervention has good fit with an individual's value system</td>
<td>How fair is [Intervention] for [people/ participants/ recipients] with [condition]?
<table>
<tr><td>Very unfair</td><td>Unfair</td><td>No opinion</td><td>Fair</td><td>Very fair</td></tr>
<tr><td>1</td><td>2</td><td>3</td><td>4</td><td>5</td></tr>
</table>
OR
There are moral or ethical consequences [behaviour i.e. to engage with] [intervention]
<table>
<tr><td>Strongly disagree</td><td>Disagree</td><td>No opinion</td><td>Agree</td><td>Strongly agree</td></tr>
<tr><td>1</td><td>2</td><td>3</td><td>4</td><td>5</td></tr>
</table>
</td>
<td>In your opinion, do you think there are moral or ethical consequences with offering one dose of the HPV vaccine to AGYW?[a]
<table>
<tr><td>Strongly disagree</td><td>Disagree</td><td>No opinion</td><td>Agree</td><td>Strongly agree</td></tr>
<tr><td>1</td><td>2</td><td>3</td><td>4</td><td>5</td></tr>
</table>
How fair is the reduced dose strategy for the AGYW?[a]
<table>
<tr><td>Very unfair</td><td>Unfair</td><td>No opinion</td><td>Fair</td><td>Very fair</td></tr>
<tr><td>1</td><td>2</td><td>3</td><td>4</td><td>5</td></tr>
</table>
</td>
</tr>
<tr>
<td>Perceived effectiveness</td>
<td>The extent to which the intervention is perceived to have achieved its objective</td>
<td>The [intervention] has improved [behaviour/ condition/ clinical outcome]:
<table>
<tr><td>Strongly disagree</td><td>Disagree</td><td>No opinion</td><td>Agree</td><td>Strongly agree</td></tr>
<tr><td>1</td><td>2</td><td>3</td><td>4</td><td>5</td></tr>
</table>
</td>
<td>To what extent do you think the HPV vaccine prevents HPV infection and cervical cancer?[b]
<table>
<tr><td>Not at all</td><td>Slight protection</td><td>No opinion</td><td>Moderate protection</td><td>Full protection</td></tr>
<tr><td>1</td><td>2</td><td>3</td><td>4</td><td>5</td></tr>
</table>
To what extent do you think the single-dose of the HPV vaccine would prevent HPV infection and cervical cancer?[a]
<table>
<tr><td>Not at all</td><td>Slight protection</td><td>No opinion</td><td>Moderate protection</td><td>Full protection</td></tr>
<tr><td>1</td><td>2</td><td>3</td><td>4</td><td>5</td></tr>
</table>
</td>
</tr>
<tr>
<td>Intervention coherence</td>
<td>The extent to which the participant understands how the intervention works</td>
<td>It is clear to me how [intervention] will help [manage/ improve] my [behaviour/ condition/clinical outcome]
<table>
<tr><td>Strongly disagree</td><td>Disagree</td><td>No opinion</td><td>Agree</td><td>Strongly agree</td></tr>
<tr><td>1</td><td>2</td><td>3</td><td>4</td><td>5</td></tr>
</table>
*Please tell us more about your views
</td>
<td>How knowledgeable are you about the single-dose strategy of the HPV vaccine?[a]
<table>
<tr><td>Very unknowledgeable</td><td>Slightly unknowledgeable</td><td>No opinion</td><td>Moderately knowledgeable</td><td>Very knowledgeable</td></tr>
<tr><td>1</td><td>2</td><td>3</td><td>4</td><td>5</td></tr>
</table>
</td>
</tr>
</table>

<table>
<tr>
<td>Self -efficacy</td>
<td>A participant's confidence that they can perform behaviour(s) required to participate in the intervention</td>
<td>How confident did you feel about [behaviour i.e. engaging with] [intervention]?
<table>
<tr><td>Very unconfident</td><td>Unconfident</td><td>No opinion</td><td>Confident</td><td>Very confident</td></tr>
<tr><td>1</td><td>2</td><td>3</td><td>4</td><td>5</td></tr>
</table>
</td>
<td>How confident are you in providing (correctly) single-doses of HPV vaccines to AGYWs?[a]
<table>
<tr><td>Very unconfident</td><td>Unconfident</td><td>No opinion</td><td>Confident</td><td>Very confident</td></tr>
<tr><td>1</td><td>2</td><td>3</td><td>4</td><td>5</td></tr>
</table>
How confident are you in explaining the benefits of the HPV vaccine to AGYWs and/ or their parents/ guardians?[b]
<table>
<tr><td>Very unconfident</td><td>Unconfident</td><td>No opinion</td><td>Confident</td><td>Very confident</td></tr>
<tr><td>1</td><td>2</td><td>3</td><td>4</td><td>5</td></tr>
</table>
How confident are you in addressing concerns that AGYWs and/ or their parents/ guardians have on the HPV vaccine?[b]
<table>
<tr><td>Very unconfident</td><td>Unconfident</td><td>No opinion</td><td>Confident</td><td>Very confident</td></tr>
<tr><td>1</td><td>2</td><td>3</td><td>4</td><td>5</td></tr>
</table>
</td>
</tr>
<tr>
<td>Opportunity costs</td>
<td>The benefits, profits or values that would have to be given up to engage with the intervention</td>
<td>[Behaviour i.e. engaging in] [intervention] interfered with my other priorities
<table>
<tr><td>Strongly disagree</td><td>Disagree</td><td>No opinion</td><td>Agree</td><td>Strongly agree</td></tr>
<tr><td>1</td><td>2</td><td>3</td><td>4</td><td>5</td></tr>
</table>
</td>
<td>NA</td>
</tr>
<tr>
<td>General acceptability</td>
<td></td>
<td>How acceptable was the [intervention] to you?
<table>
<tr><td>Completely unacceptable</td><td>Unacceptable</td><td>No opinion</td><td>Acceptable</td><td>Completely acceptable</td></tr>
<tr><td>1</td><td>2</td><td>3</td><td>4</td><td>5</td></tr>
</table>
</td>
<td>In your opinion, how acceptable do you think it is to provide a single-dose of the HPV vaccine to AGYW?[a]
<table>
<tr><td>Very unacceptable</td><td>Unacceptable</td><td>No opinion</td><td>Acceptable</td><td>Very acceptable</td></tr>
<tr><td>1</td><td>2</td><td>3</td><td>4</td><td>5</td></tr>
</table>
</td>
</tr>
</table>

[a] Questions adapted from the TFA generic questionnaire | [b]Additional questions related to TFA constructs that were deemed important in influencing the acceptability of the single-dose schedule of HPV vaccination in Kenya among HCPs

Acceptability of the Reduced Dose Strategy of HPV Vaccine

Thank you for taking the time to participate in this study and for completing this survey.

The Kenya Ministry of Health (MoH) recommendation for HPV vaccination is a two-dose schedule 0 and 6 months similar to the World Health Organization (WHO) Recommendations. Kenya currently has a national HPV vaccine program that was launched in 2019 based on evidence from a school-based vaccination for 9-10 years. GAVI has supported two HPV vaccination demonstration projects from 2013-2015 and 2016-present in Kitui county (outside of our proposed study areas). Both demonstration projects used the quadrivalent HPV 16/18/6/11 vaccine in a school-based strategy that targeted girls aged 9-10 years, and the first achieved 85% coverage.

Considering the recent endorsement of the WHO or the reduced dose strategy of the HPV vaccine for adolescent girls and young women (AGYWs), we are interested in your perceptions of how acceptable you think the reduced dose strategy is and what would make HPV vaccination a sustainable component of cervical cancer prevention among AGYW in our communities.

All your responses will be de-identified and this component of the study will not require any recording.

Do you have any questions please feel free to email or call the project PI Dr. Lynda Oluoch at lynda@pipsthika.org or 0736464299

Phone number

____
(Please note that this number will not be shared outside the KEMRI study team)

Part I: Demographics Information

Date

____

1. Modality used to complete the questionnaire

◯ In-person/ guided survey
◯ Phone
◯ Computer/ Laptop
◯ Other
(Please clarify how you are completing this survey)

1. a. Please specify

____

2. Gender

◯ Male
◯ Female

3. Age (years)

____

4. Religion

◯ Christian
◯ Muslim
◯ Hindu
◯ Other

4. a. Please specify

____

5. County

◯ Mombasa
◯ Kwale
◯ Kilifi
◯ Tana River
◯ Lamu
◯ Taita/Taveta
◯ Garissa
◯ Wajir
◯ Mandera
◯ Marsabit
◯ Isiolo
◯ Meru
◯ Tharaka-Nithi
◯ Embu
◯ Kitui
◯ Machakos
◯ Makueni
◯ Nyandarua
◯ Nyeri
◯ Kirinyaga
◯ Murang'a
◯ Kiambu
◯ Turkana
◯ West Pokot
◯ Samburu
◯ Trans Nzoia
◯ Uasin Gishu
◯ Elgeyo/Marakwet
◯ Nandi
◯ Baringo
◯ Laikipia
◯ Nakuru
◯ Narok
◯ Kajiado
◯ Kericho
◯ Bomet
◯ Kakamega
◯ Vihiga
◯ Bungoma
◯ Busia
◯ Siaya
◯ Kisumu
◯ Homa Bay
◯ Migori
◯ Kisii
◯ Nyamira
◯ Nairobi City
(Please pick one)

6. Name of the facility where participant works

____

7. Participant Job Title

◯ Nurse
◯ Clinical Officer
◯ Medical Officer
◯ Pharmacist Technologist
◯ Pharmacist
◯ Hospital Director
◯ Other

7. a. Please specify

____

8. Duration at that job	__________________________
8. a. The duration at the job mentioned above is in	◯ Days<br>◯ Weeks<br>◯ Months<br>◯ Years
9. Level of the health system where the participant is employed	◯ National Referral (level 6)<br>◯ County Referral (level 5)<br>◯ County hospitals (level 4)<br>◯ Health center (level 3)<br>◯ Health dispensaries (level 2)<br>◯ Community Facilities (level 1)<br>◯ Other
9. a. Please specify	__________________________
10. Geographic location of the facility	◯ Rural<br>◯ Urban<br>◯ Other
10. a. Please specify	__________________________

Part II: Questions on the Acceptability of the HPV vaccine reduced dose strategy

1.General acceptability<br>In your opinion, how acceptable do you think it is to provide a single dose of the HPV vaccine to AGYW?	◯ Very unacceptable<br>◯ Unacceptable<br>◯ Neutral<br>◯ Acceptable<br>◯ Very acceptable
2. Burden	
2.1 How much effort does it take you to provide one dose of the HPV vaccine to AGYW?	◯ No effort at all<br>◯ A little effort<br>◯ Neutral<br>◯ A lot of effort<br>◯ Huge effort
2.2. In your opinion, how much time does it/ will it take you to provide a single dose of the HPV vaccine to AGYW?	◯ No time at all<br>◯ Little time<br>◯ No opinion<br>◯ A lot of time<br>◯ A huge amount of time
2.3. In your opinion, how much resource does it/ will it take you to provide a single dose of the HPV vaccine to AGYW?	◯ No resource at all<br>◯ Little resource<br>◯ No opinion<br>◯ A lot of resources<br>◯ Huge resources

2.4 How much effort does it take to incorporate the single dose into the existing workflow at your clinic/ facility	◯ No effort at all<br>◯ A little effort<br>◯ No opinion<br>◯ A lot of effort<br>◯ Huge effort
3. Ethicality	
3.1. In your opinion, do you think there are moral or ethical consequences with offering one dose of the HPV vaccine to AGYW?	◯ Strongly disagree<br>◯ Disagree<br>◯ Neutral<br>◯ Agree<br>◯ Strongly agree
3.2. How fair is the reduced dose strategy for the AGYWs?	◯ Very unfair<br>◯ Unfair<br>◯ No opinion<br>◯ Fair<br>◯ Very fair
4. Perceived effectiveness	
4.1 To what extent do you think the HPV vaccine prevents HPV infection and cervical cancer?	◯ Not at all<br>◯ Slight protection<br>◯ Not really |No opinion<br>◯ Somehow Moderate protection<br>◯ Very much Full protection
4.2 To what extent do you think the single dose of the HPV vaccine would prevent HPV infection and cervical cancer?	◯ Not at all<br>◯ Slight protection<br>◯ Not really No opinion<br>◯ Somehow Moderate protection<br>◯ Very much Full protection
5. Self-efficacy	
5.1 How confident are you in providing (administering correctly) single doses of HPV vaccines to AGYWs?	◯ Very unconfident<br>◯ Slightly unconfident<br>◯ No opinion<br>◯ Moderately confident<br>◯ Very confident
5.2 How confident are you in explaining the benefits of the HPV vaccine to AGYWs and/ or their parents/ guardians?	◯ Very unconfident<br>◯ Slightly unconfident<br>◯ No opinion<br>◯ Moderately confident<br>◯ Very confident
5.3 How confident are you in addressing concerns that AGYWs and/ or their parents/ guardians have on the HPV vaccine?	◯ Very unconfident<br>◯ Slightly unconfident<br>◯ No opinion<br>◯ Moderately confident<br>◯ Very confident
6. In your opinion, what factors enable your ability to confidently deliver a single dose of the HPV vaccine?	______________________________<br>(What has made you/ makes you confident?)

7. What limits your confidence in delivering a single dose of the HPV vaccine?

____
(What has made you/ makes you less confident?)

8. Knowledge

8.1. How knowledgeable are you about the single-dose strategy of the HPV vaccine?

- ◯ Very unknowledgeable
- ◯ Slightly unknowledgeable
- ◯ No opinion
- ◯ Moderately knowledgeable
- ◯ Very knowledgeable

8.2. What do you know about the single-dose strategy of the HPV vaccine?

____

8.3. How did you learn about the single-dose strategy of the HPV vaccine?

____

9. What are your beliefs regarding the single-dose strategy of the HPV vaccine?

____

General Comments

Other comments?

____

Supplementary Tables

Supplementary Table 1: Correlation between the reported general acceptability and the computed acceptability varying constructs used to represent relevant domains (with all variables treated as continuous variable)

Item	Components	Distribution of Score Across Counties Mean (SD)					Spearman Correlation	
		Kiambu	Kisumu	Nairobi	Overall	P-value[1]	Coefficient	P-value[2]
**General acceptability (reported)**	NA	4.01 (1.30)	3.74 (1.33)	3.65 (1.22)	3.79 (1.29)	0.1		
**Acceptability mean score1**	• Effort<br>• Moral consequence<br>• Confidence<br>• Effectiveness<br>• Knowledge	3.76 (0.51)	3.71 (0.52)	3.70 (0.51)	3.72 (0.52)	0.69	0.052	0.31
**Acceptability mean score2**	• Time<br>• Moral consequence<br>• Confidence<br>• Effectiveness<br>• Knowledge	3.79 (0.49)	3.74 (0.53)	3.74 (0.51)	3.75 (0.51)	0.69	0.043	0.40
**Acceptability mean score3**	• Resource<br>• Moral consequence<br>• Confidence<br>• Effectiveness<br>• Knowledge	3.77 (0.48)	3.68 (0.52)	3.69 (0.52)	3.71 (0.51)	0.35	0.04	0.43
**Acceptability mean score4**	• Effort<br>• Fairness<br>• Confidence<br>• Effectiveness<br>• Knowledge	4.10 (0.59)	3.98 (0.62)	3.95 (0.51)	4.0 (0.58)	0.15	0.18	**<0.05 ***
**Acceptability mean score5**	• Time<br>• Fairness<br>• Confidence<br>• Effectiveness<br>• Knowledge	4.13 (0.54)	4.01 (0.62)	3.99 (0.52)	4.04 (0.57)	0.16	0.19	**<0.05 ***

**Acceptability mean score6**	• Resource<br>• Fairness<br>• Confidence<br>• Effectiveness<br>• Knowledge	4.11 (0.54)	3.95 (0.62)	3.93 (0.55)	3.99 (0.58)	0.055	0.17	**<0.05 ***

[1]P-value comparing scores across sites [2]P-value of the correlation coefficient *P-value <0.05

<u>Supplementary Table 2</u>: Output from the regression model assessing the association between constructs of acceptability and the reported general acceptability.

**Construct**	**Using the moral consequence construct for the ethicality domain**			**Using fairness construct for the ethicality domain**			**Accounting for all relevant constructs and domains of acceptability**		
	**Beta**	**95% CI[1]**	**P-value**	**Beta**	**95% CI[1]**	**P-value**	**Beta**	**95% CI[1]**	**P-value**
Effort*	-0.07	-0.21, 0.07	0.3	-0.07	-0.21, 0.06	0.3	-0.03	-0.19, 0.13	0.7
Moral consequence	0.05	-0.07, 0.16	0.4				0.06	-0.06, 0.17	0.3
Fairness				0.22	0.09, 0.35	**0.001***	0.23	0.10, 0.36	**<0.001***
Perceived effectiveness of 1-dose	0.12	-0.03, 0.28	0.12	0.05	-0.10, 0.21	0.5	0.08	-0.08, 0.24	0.3
Confidence giving 1-dose	0.03	-0.10, 0.16	0.6	-0.01	-0.14, 0.12	>0.9	0	-0.13, 0.13	>0.9
Knowledge on 1-dose	-0.06	-0.17, 0.06	0.3	-0.04	-0.16, 0.07	0.5	-0.06	-0.17, 0.06	0.3
Time							-0.02	-0.30, 0.25	0.9
Resources							-0.07	-0.25, 0.11	0.5
Effort to integrate 1-dose							0	-0.18, 0.18	>0.9

[1]CI = Confidence Interval *P-value< 0.05

Supplementary Table 3: Correlation between the general acceptability and the different constructs of acceptability (Treated as categorical variables)

		**Polychronic Correlation Coefficient**
**Burden#**	Effort to give 1 dose	-0.031
	Time to give 1 dose	0.013
	Resource to give 1 dose	-0.03
	Effort to integrate 1 dose into Existing work	-0.012
**Ethicality**	Moral consequences of giving 1 dose	-0.04
	Fair to give 1 dose	0.29
**Perceived Effectiveness**	Effectiveness of the HPV vaccine	0.16
	Effectiveness of 1 dose the HPV vaccine	0.15
**Self-Efficacy**	Confidence giving 1 dose	0.09
	Confidence explaining benefits of the vaccine	0.06
	Confidence addressing concerns on the vaccine	0.05
**Intervention Coherence**	Knowledge on 1 dose	0.006

*Excluded responses (n=11) that were missing data for acceptability domains and constructs of interest

The score of constructs of the burden domain were reversed before conducting the correlation analysis

Supplementary Table 4: Determinants of acceptability among HCPs. Findings of a multivariate linear regresion model (without interraction)

	**Beta**	**95% CI[1]**	**p-value**
Gender			
Male (Ref)	—	—	
Female	0.05	-0.28, 0.38	0.8
Age	-0.01	-0.04, 0.02	0.5
religion			
Christian	—	—	
Muslim	-0.07	-1.0, 0.89	0.9
Other	0.81	-0.70, 2.3	0.3
Job Title			
Nurse (Ref)	—	—	
Clinical Officer	-0.01	-0.45, 0.44	>0.9
Medical Officer	0.24	-0.41, 0.90	0.5
Pharmacist Technologist	0.46	-0.21, 1.1	0.2
Pharmacist	0.25	-0.66, 1.2	0.6
Hospital Director	-0.25	-1.8, 1.3	0.8
Other	0.37	0.01, 0.73	**0.044***
Job duration (Months)	0	0.00, 0.00	0.7
Facility Level			
Facility_level.L	0.39	-0.24, 1.0	0.2

Facility_level.Q	0.07	-0.49, 0.62	0.8
Facility_level.C	0.3	-0.26, 0.86	0.3
Facility_level^4	-0.34	-0.88, 0.21	0.2
Facility_level^5	-0.03	-0.48, 0.43	>0.9
Facility_level^6	-0.13	-0.46, 0.19	0.4
Geographic location			
Rural (Ref)	—	—	
Urban	-0.12	-0.52, 0.28	0.6
Other	0.09	-1.8, 2.0	>0.9
County			
Kiambu (Ref)	—	—	
Kisumu	-0.19	-0.55, 0.17	0.3
Nairobi City	-0.36	-0.76, 0.04	0.077

[1] CI = Confidence Interval *P-value <0.05

Model no interraction with complete data only (n=356)

Supplementary Table 5: Determinants of acceptability among HCPs. Findings of a multivariate linear regresion model (with interraction between referral level and county as well as job title and job duration)[#]

	**Beta**	**95% CI[1]**	**p-value**
**Gender**			
Male (Ref)	—	—	
Female	-0.02	-0.35, 0.31	>0.9
**Age**	-0.01	-0.04, 0.01	0.3
**Religion**			
Christian (Ref)	—	—	
Muslim	-0.09	-1.1, 0.87	0.9
Other	0.85	-0.67, 2.4	0.3
**Job title**			
Nurse (Ref)	—	—	
Clinical Officer	0.3	-0.36, 0.95	0.4
Medical Officer	-0.14	-1.2, 0.90	0.8
Pharmacist Technologist	0.66	-0.34, 1.7	0.2
Pharmacist	0.67	-0.93, 2.3	0.4
Hospital Director	-1	-3.2, 1.2	0.3
Other	0.3	-0.20, 0.81	0.2
Job duration (months)	0	0.00, 0.00	0.5
**Facility level**			
Facility level.L	17	1.4, 33	**0.033**
Facility level.Q	-18	-33, -2.2	**0.025**
Facility level.C	14	2.1, 27	**0.022**
Facility level ^4	-6.1	-12, 0.03	0.051
facility_level^5	5.2	1.2, 9.3	**0.011**
facility_level^6	-0.24	-0.67, 0.20	0.3
**Geographic location**			

	**Beta**	**95% CI[1]**	**p-value**
**County**			
Kiambu (Ref)	—	—	
Kisumu	-0.19	-0.57, 0.19	0.3
Nairobi City	-0.43	-0.84, -0.02	**0.039**
**Job title * Job duration (months)**			
Clinical Officer * Job duration	0	-0.01, 0.00	0.3
Medical Officer * Job duration	0	-0.01, 0.02	0.4
Pharmacist Technologist * Job duration	0	-0.02, 0.01	0.5
Pharmacist * Job duration	0	-0.02, 0.01	0.6
Hospital Director * Job duration	0.03	-0.03, 0.09	0.3
Other * Job duration	0	0.00, 0.01	0.9
**Facility level * geographic location**			
Facility level.L * Urban	-17	-33, -1.1	**0.037**
Facility level.Q * Urban	18	2.3, 34	**0.025**
Facility level.C * Urban	-14	-26, -1.7	**0.026**
Facility level ^4 * Urban	5.5	-0.50, 12	0.072
Facility level ^5 * Urban	-5.3	-9.3, -1.3	**0.009**
Facility level ^6 * Urban			
Facility level.L * Other	3.9	-4.2, 12	0.3
Facility level.Q * Other			
Facility level.C * Other			
Facility level ^4 * Other			
Facility level ^5 * Other			
Facility level ^6 * Other			

Rural (Ref)	—	—	
Urban	5	0.71, 9.3	**0.022**
Other	-1	-3.7, 1.7	0.5

[#]Complete data (n=356)

[1] CI = Confidence Interval

Numbers in bold= p-values < 0.05

<u>Supplementary Table 6</u>: Model selection: Checking the best fitting model

**Model**	**Description**	**AIC**
model2	Linear multiregression model without interraction all data available (n=385)	1253.8
model2b	Linear multiregression model without interraction no NAs (n=356)	***1221.6***
model3	Linear multiregression model with interraction between job title and dutation as well as referral level and geographic location (n=385)	1262.3
model3b	Linear multiregression model with interraction between referral level and geographic location (n=385)	1254.3
model4	Linear multiregression model with interraction between job title and dutation as well as referral level and geographic location (n=356)	***1230.4***
model5	Multivariate poisson regression model without interraction (n=385)	**1383.7**
model6	Multivariate poisson regression model with interraction between job title and dutation as well as referral level and geographic location (n=385)	1400.5

Supplementary Figures

Supplementary Figures 1:: Distribution of responses on domains of acceptability overall and across counties

a. **General Acceptability**

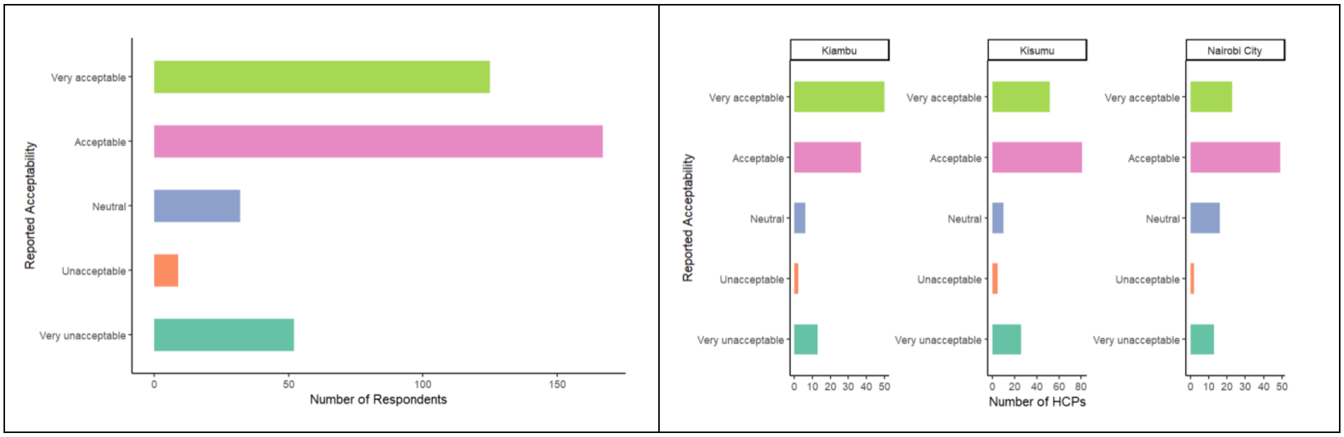

Constructs of Acceptability

b. **Burden ie Effort required to participate in the EBI**

Effort to give 1 dose

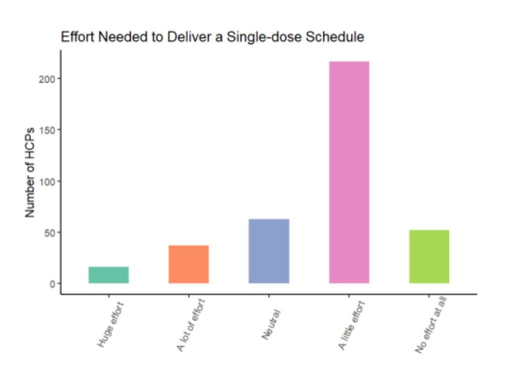

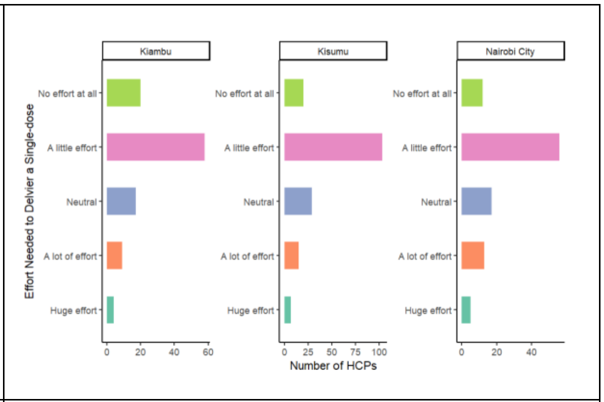

Time to give 1 dose

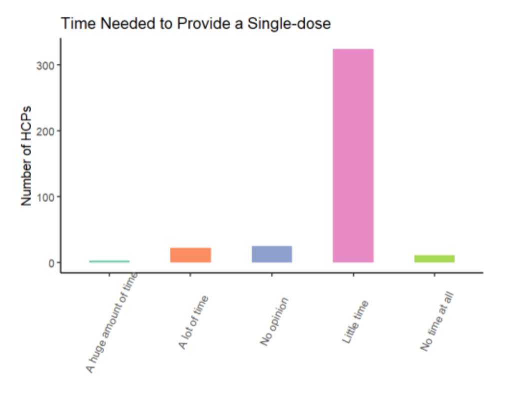

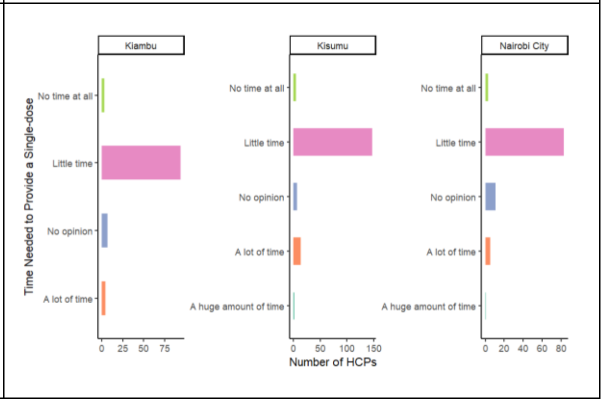

<table>
<tr>
<td>Resource to give 1 dose</td>
<td>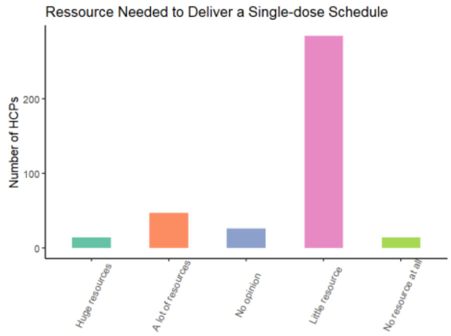
</td>
<td>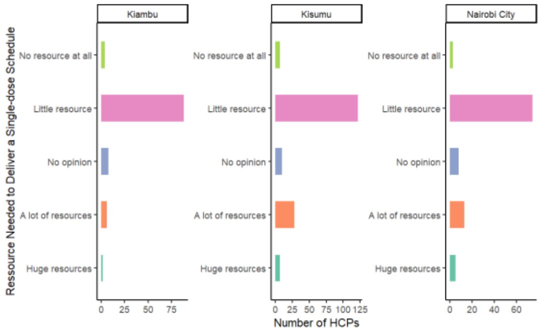
</td>
</tr>
<tr>
<td>Effort to integrate 1 dose into existing work</td>
<td>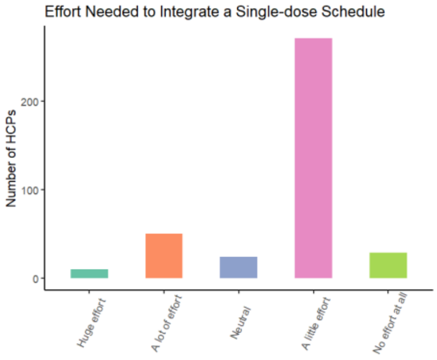
</td>
<td>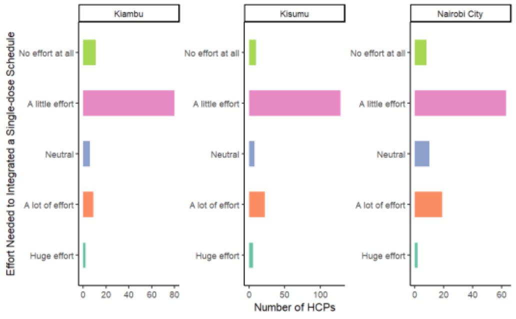
</td>
</tr>
</table>

c. Ethicality ie Fair or moral consequences

<table>
<tr>
<td>Moral Conseque nce of Giving 1 dose</td>
<td>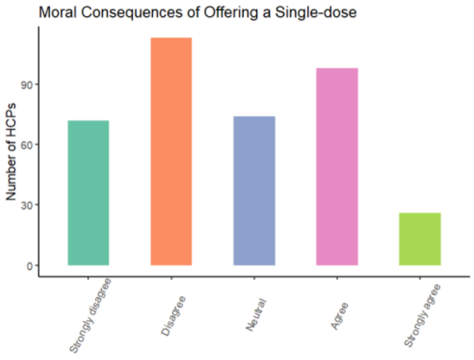
</td>
<td>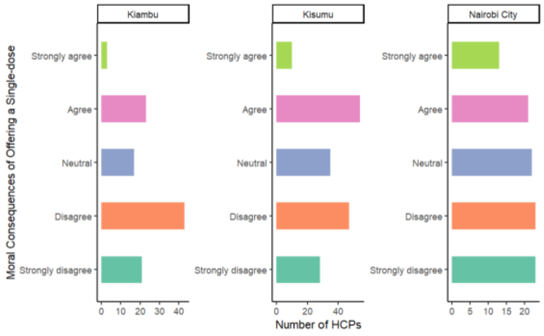
</td>
</tr>
<tr>
<td>Fairness</td>
<td>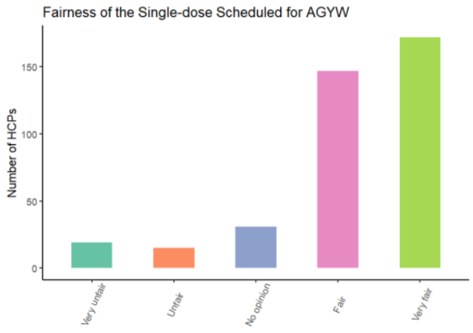
</td>
<td>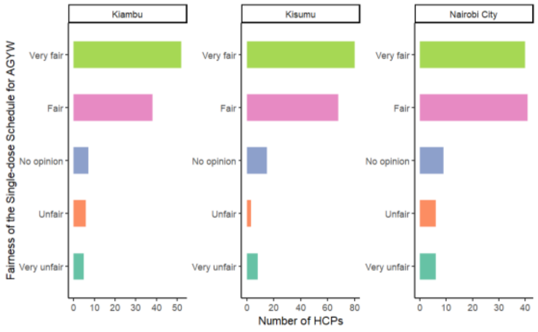
</td>
</tr>
</table>

d. Perceived Effectiveness ie Extent to which the EBI is perceived to achieve its objective

Effectiveness of the HPV vaccine

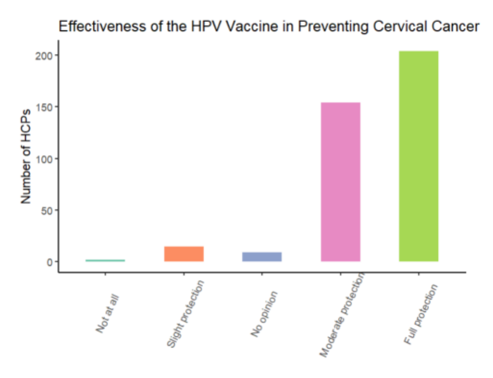

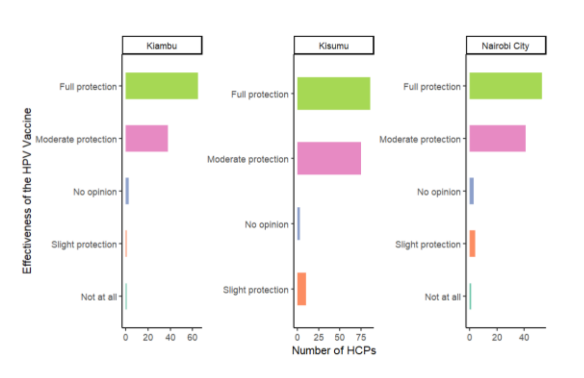

Effectiveness of 1 dose

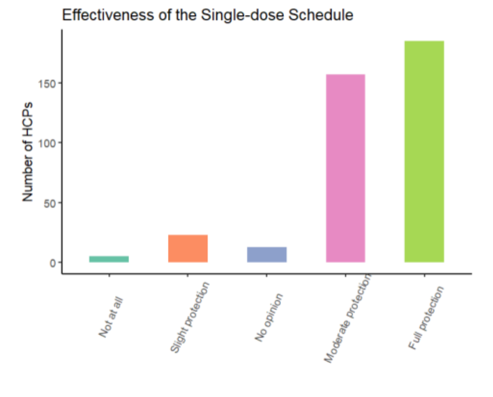

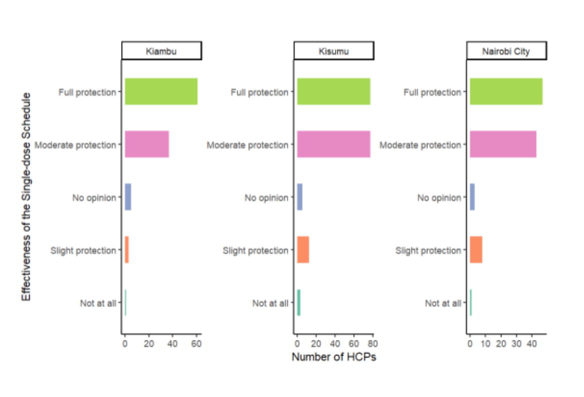

e. Self-Efficacy – confidence participating in the EBI

Confidence providing 1 dose

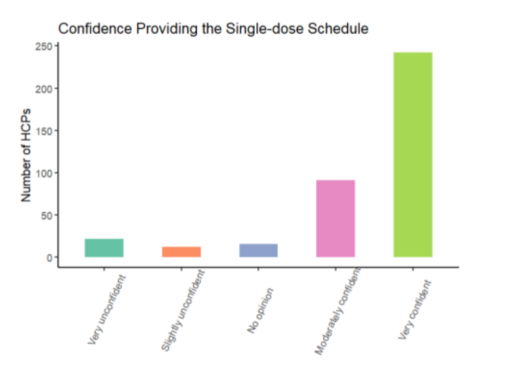

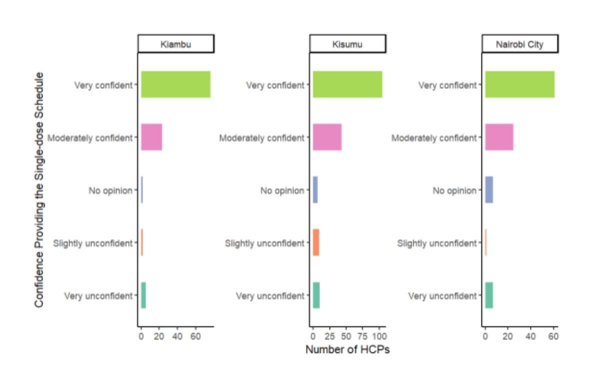

Confidence explaining benefits of the vaccine

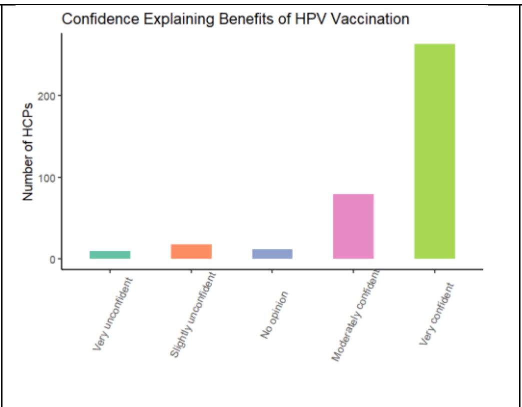

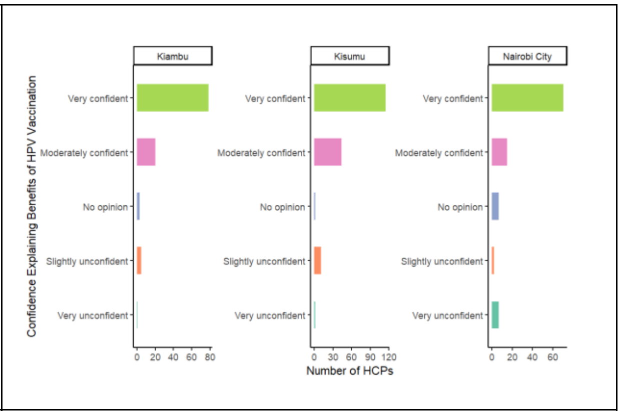

Confidence addressing concerns on HPV vacine

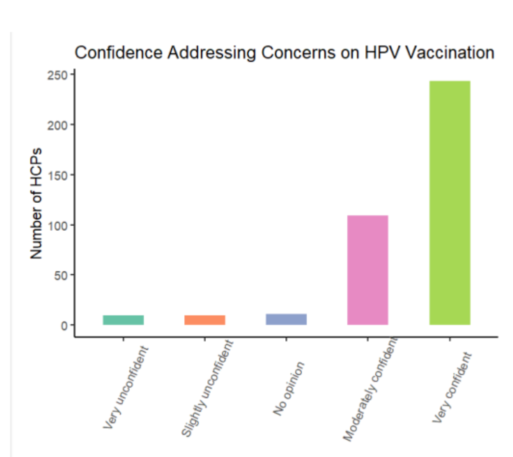

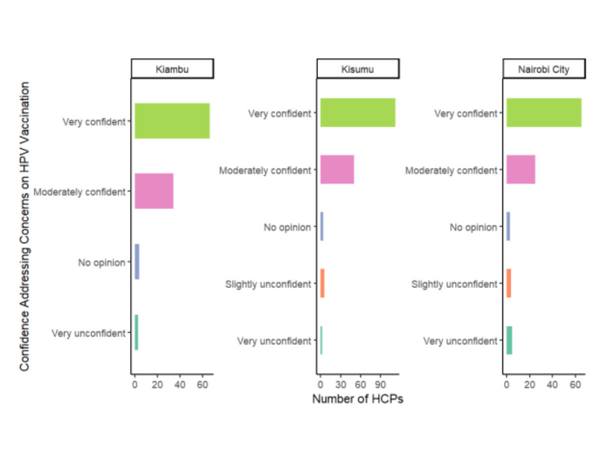

f. Knowledge/ Intervention coherence (ie understanding of how the EBI works)

Knowledge on single-dose

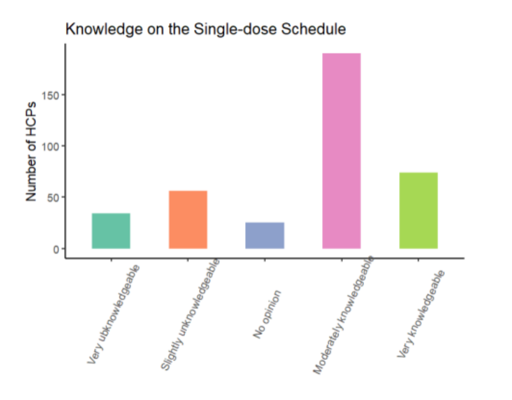

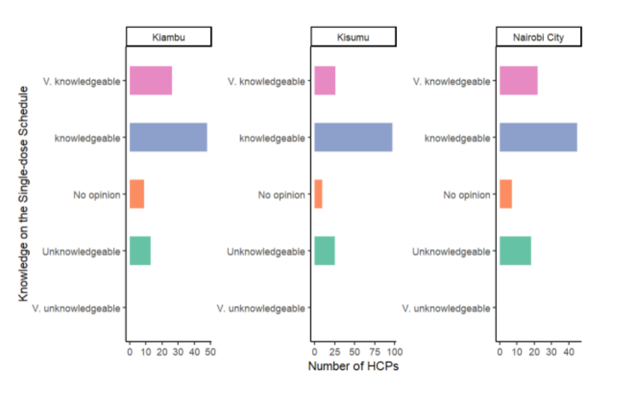

Supplementary Figure 2: Model Selection: Assessing the linearity assumption for the linear regression model

	**Model 2b: Linear multiregression model without interraction no NAs (n=356)**	**Model 4 : Linear multiregression model with interraction between job title and dutation as well as referral level and geographic location (n=356)**
**Plot true vs precited values**	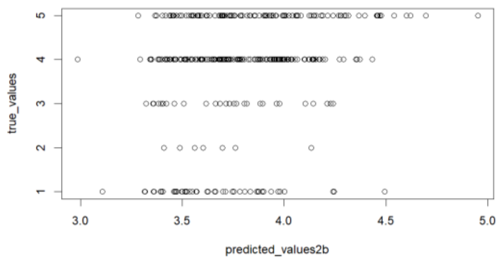	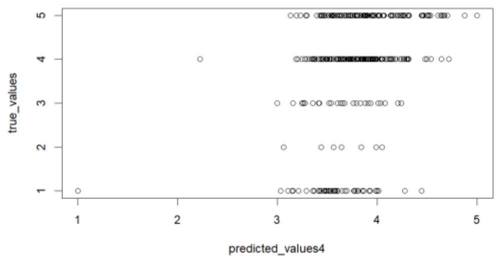
**Plot of residuals vs predicted values**	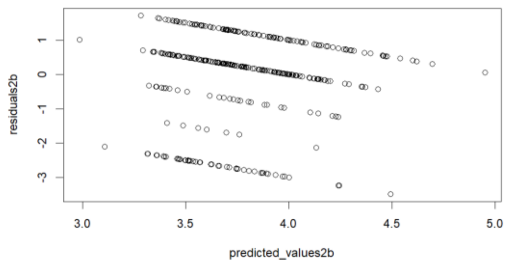	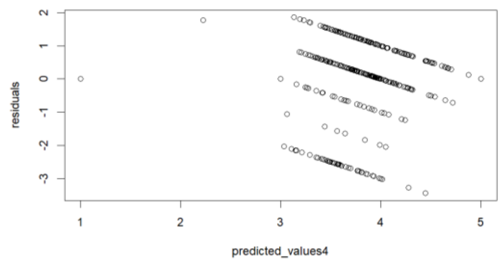

Residuals seem to be following a clear pattern and are not quite random in nature so a linear model is not the best model for this data. Thus a logistics regression model with the lowest AIC is more appropriate.

<u>Supplementary Figure 3</u>: Comparison of estimates of determinants of acceptability among HCPs. Findings of a multivariate logistic regression with and without the fairness construct included in the model, reporting incidence rate ratios (IRR) with 95% Confidence Intervals (CI)

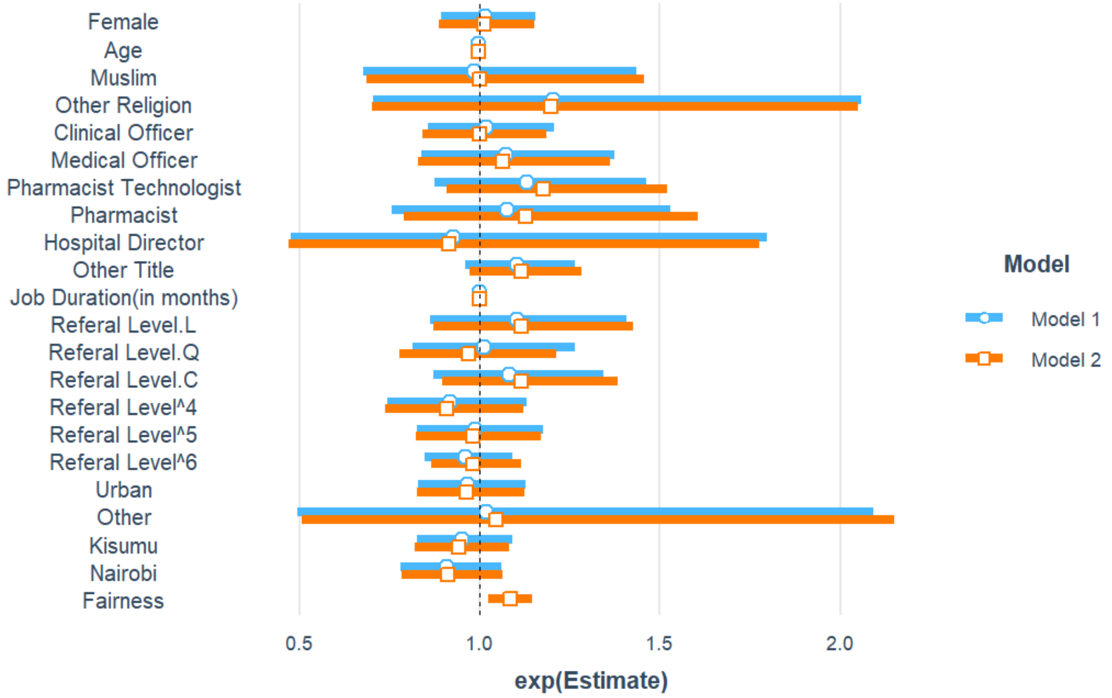

Model 1: outputs from the multivariate logistic regression reported on table 5 (without the fairness score)
Model 2: outputs from the multivariate logistic regression with the fairness construct

<u>Supplementary Figure 4</u>: Determinants of acceptability among HCPs. Findings of a multivariate poisson regresion reporting incidence rate ratios (IRR) with robust standard errors (HC1)

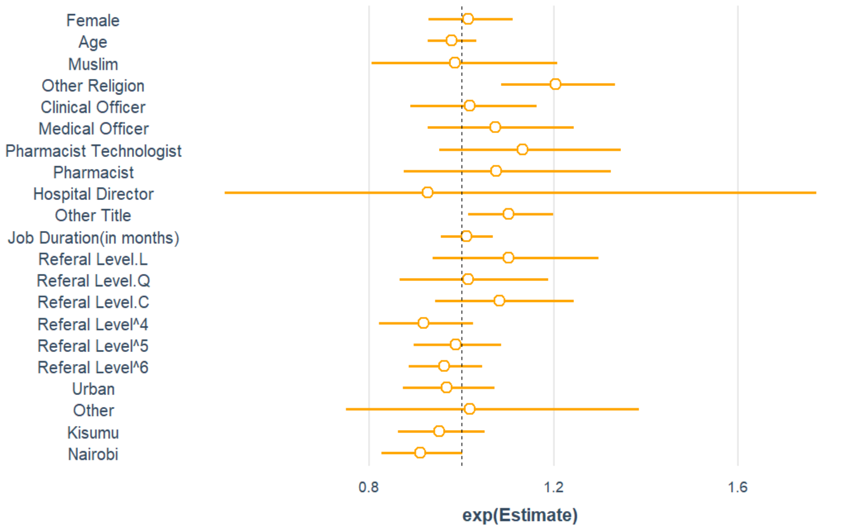

Supplement: lgae031_Supplementary_Data [file lgae031_supplementary_data.pdf]
